# Optimizing Clinical Management of COVID-19: A Predictive Model for Unvaccinated Patients Admitted to ICU

**DOI:** 10.3390/pathogens14030230

**Published:** 2025-02-27

**Authors:** Ahmed Farhan, Khouloud Ayed, Sara Zayati, Rym Akrout, Akram Dlala, Amal Abouda, Nesrine Zoghlami, Halima Mahjoubi, Iheb Labbane, Asma Gati

**Affiliations:** 1 Laboratory of Genetics, Immunology, and Human Pathologies, Faculty of Sciences of Tunis, University Tunis El Manar, Tunis 2092, Tunisia; ahmed.farhan@fst.utm.tn (A.F.); khouloud.ayed@etudiant-fst.utm.tn (K.A.); sarra.zayati@etudiant-fst.utm.tn (S.Z.); rym.akrout@etudiant-fst.utm.tn (R.A.); akram.dlala@etudiant-fst.utm.tn (A.D.); 2 Department of Anesthesiology and Intensive Care, Military Hospital of Tunis, Tunis 1008, Tunisia; amalabouda@gmail.com (A.A.); iheb.labbene@fmt.utm.tn (I.L.); 3 Laboratoire de Télédétection et Systèmes d’Information à Référence Spatiale (LTSIRS), Tunis 1002, Tunisia; nesrine.zoghlami@ipeiem.utm.tn; 4 Laboratoire de Biophysique et Technologies Médicales, Institut Supérieur des Technologies Médicales de Tunis (ISTMT), Tunis 1006, Tunisia; halima.mahjoubi@utm.tn

**Keywords:** cytokines (IL-6; TNF-α), comorbidities, COVID-19 outcomes, unvaccinated patients, immunological markers, predictive modeling, recovery and mortality rates

## Abstract

This study investigates the impact of immunological and clinical factors on COVID-19 outcomes among unvaccinated individuals. A cohort of 42 unvaccinated patients admitted to an intensive care unit was analyzed, focusing on age, comorbidities, inflammatory cytokines (IL-6, TNF-α), and anti-SARS-CoV-2 spike protein antibody levels (IgG) to assess their influence on hospital stay duration, recovery time, complications, and mortality rates. The findings revealed that advanced age, cardiovascular disease, and elevated pro-inflammatory cytokines significantly heightened the risks of severe complications and mortality. Conversely, low IgG levels correlated with prolonged hospital stays and slower recovery. Multivariate analysis identified high IL-6 and TNF-α levels as strong predictors of adverse outcomes. This research emphasizes the need for the early monitoring of cytokines and targeted management strategies to mitigate the impact of COVID-19, especially among high-risk unvaccinated populations.

## 1. Introduction

The COVID-19 pandemic continues to impose a significant burden on global healthcare systems. As of 2023, ICU admission rates among unvaccinated COVID-19 patients remain disproportionately high, with mortality rates ranging between 30% and 50% depending on comorbidity profiles [1,2]. Additionally, studies indicate that patients with preexisting cardiovascular disease have a 2.5-fold increased risk of severe complications [3]. These factors emphasize the ongoing public health challenge posed by severe COVID-19 cases, particularly in high-risk unvaccinated populations. A number of studies identified that comorbid patients, especially those suffering from cardiovascular diseases, diabetes, and respiratory conditions, have a notably higher risk of severe complications associated with COVID-19, further prolonging their hospitalization and increasing their rate of mortality and recovery time [2]. These comorbidities interact in a complicated way, enhancing an acute impact of SARS-CoV-2 infection and developing so-called long COVID—a state in which symptoms and complications of an illness stand out even after its acute phase has passed [3,4].

Understanding how SARS-CoV-2 interacts with host immune responses and preexisting comorbidities is critical in predicting clinical outcomes and designing personalized treatment strategies. Recent studies have established that immune dysregulation in severe COVID-19 cases is driven by the excessive production of IL-6 and TNF-α, leading to a hyperinflammatory state known as the cytokine storm [5]. Additionally, patients with underlying metabolic and cardiovascular conditions exhibit a maladaptive immune response, further exacerbating disease severity [6]. The presence of elevated IFN-γ and TNF-α levels has been strongly linked to increased ICU admissions and prolonged hospitalization periods [7]. Immune dysfunction is pivotal in the development of severe COVID-19, as evidenced by the common observation of higher pro-inflammatory cytokine levels, including IL-6, TNF-α, and IFN-γ, in such cases [5,6]. These biomarkers do not only represent the physiological response of the body to viral infections but are often influenced by the underlying disease, which can precipitate hyperimmune responses, cytokine storms, organ damage, and, in some cases, death [7]. Of particular note, elevated plasma levels of IL-6 have been associated with more severe respiratory disease and increased need for hospitalization [8]. Similarly, systemic inflammatory molecules such as TNF-α and IFN-γ have also been associated with a poor prognosis in these comorbid conditions of diabetes and cardiovascular diseases [9,10].

In recent years, machine learning (ML) and artificial intelligence (AI) techniques have been increasingly used to stratify COVID-19 patients based on immune biomarkers and clinical factors. Tang et al. (2024) demonstrated that AI-based prediction models incorporating inflammatory markers (IL-6, TNF-α, IFN-γ) significantly improve risk assessment for severe cases. Similarly, Heydari et al. (2023) conducted a large-scale ICU study in an unvaccinated cohort and found that age, cytokine levels, and preexisting cardiovascular disease were key determinants of mortality. However, while these studies have contributed valuable insights, there is still a gap in integrating cytokine profiling with a predictive model tailored specifically for unvaccinated ICU patients [11,12].

Our study seeks to bridge this gap by developing a predictive model that combines cytokine levels (IL-6, TNF-α, IFN-γ), antibody responses (IgG), and comorbid conditions to assess the likelihood of complications, prolonged hospital stays, and mortality in severely ill, unvaccinated COVID-19 patients [13]. These approaches make use of multi-omics datasets that further enhance the stratification of patients, hence allowing practitioners to predict high-risk cases and manage them with a great deal of accuracy [14]. Multi-omics research has suggested that inflammatory and metabolic profiling might be integrated into identifying important pathways associated with immune dysregulation, thereby enhancing clinicians’ capabilities to predict disease severity and mortality more accurately [15]. Such models are especially useful in patients with comorbidities, in whom the usual assessment technique often addresses the complex interaction of such risk factors poorly [16].

In the absence of vaccination within the cohort studied, these immune responses have been unprovoked, making the individuals more vulnerable to severe inflammatory responses. The research findings also indicate that, generally, the unvaccinated maintain a high degree of inflammatory markers such as IL-6 and TNF-α, which further lead to the development of complications in susceptible populations with comorbid conditions [16]. The absence of vaccination eliminates an important protective factor and worsens the course of COVID-19 in patients with chronic conditions, but especially in those with cardiovascular or metabolic diseases [17,18].

Presently, this research work, following previous studies, represents the analysis of outcomes in unvaccinated COVID-19 cases. We sought to develop a predictive model of clinical outcomes, including duration of stay in the hospital, recovery time, and mortality, using immunological and biochemical markers. We studied markers like IL-6, TNF-α, and IgG in relation to their effect on disease progression and long-term health outcomes in a cohort of unvaccinated patients with diverse comorbidities. The currently followed approach emphasizes that correct, evidence-based strategies for identifying high-risk patients are urgently needed to enable appropriate allocation of resources and the planning of personalized treatment pathways within the clinical setting itself [19,20].

There have been some investigations of the clinical outcomes of unvaccinated COVID-19 patients who were admitted to ICUs, such as those by Heydari et al. (2023) and Tang et al. (2024) [11,12]. There are some limitations to these investigations that our investigation tries to address. Heydari et al. (2023) concentrated on overall clinical determinants of ICU results in unvaccinated patients without including cytokine profiling as a predictive marker of disease severity and mortality. Conversely, Tang et al. (2024) established machine learning models for COVID-19 mortality prediction based on gigantic patient datasets without an extensive examination of cytokine interaction and immune dysregulation. Our research bridges this important divide by creating a cytokine-based predictive model for unvaccinated ICU patients, wherein IL-6 and TNF-α were identified as significant biomarkers of disease progression. In contrast to earlier research, we propose a cytokine-to-antibody ratio stratification system for categorizing patients into low-, moderate-, and high-risk groups, enabling early intervention and better patient management. Moreover, we present clinical validation of cytokine cutoffs for complications, extended ICU stay, and mortality—results not directly addressed in previous research. Our results move the field of personalized medicine forward by presenting a focused strategy toward risk stratification and early identification, ultimately allowing for more efficient ICU management protocols for unvaccinated COVID-19 high-risk patients [11,12].

## 2. Methods

### 2.1. Study Design and Population

A retrospective observational approach was applied in the current study, reviewing clinical and immunological data among COVID-19 patients. The electronic health records of patients were collected. In total, 42 unvaccinated patients diagnosed with severe COVID-19 and admitted to the intensive care unit of the Military Hospital of Tunis, from October 2020 to April 2021, formed the cohort of the current study. All cases presented with a positive determination of COVID-19 by PCR and were aged 40 years or older, with complete clinical and laboratory data included. This will also include patients with non-COVID-19 immunosuppression states and those without specific major immunological markers.

### 2.2. Data Collection and Variables

Patient data included demographic characteristics, comorbidities, immunological markers, biochemical markers, and health outcomes. The primary variables collected were the following:

- Demographics and comorbidities: age, gender, blood group, lifestyle factors (e.g., smoking, alcohol consumption), and comorbidities (e.g., cardiovascular disease, diabetes, respiratory conditions).
- Complications and outcomes: complications (thrombosis, myocarditis, pulmonary embolism), length of hospital stay, mortality, and time to recovery were recorded from the patient files.

### 2.3. Immunological and Biochemical Analysis

- Immunological marker assessment: Blood samples were taken from all the patients within 24 h of admission to the ICU for uniformity of biomarker analysis. Serum concentrations of IL-6, TNF-α, IFN-γ, IL-2, IL-4, and anti-SARS-CoV-2 spike protein IgG were analyzed by enzyme-linked immunosorbent assay (ELISA) kits from Bioassay Technology Laboratory (Shanghai, China) and Sunlong Biotech Co., Ltd. (Hangzhou, China) following the manufacturer’s guidelines. For more accurate measurements, all ELISA assays were conducted in duplicate, and average values were used for analysis.

During sample preparation, venous blood samples were collected in EDTA tubes (Sunlong Biotech Co., Ltd., Hangzhou, China) and immediately centrifuged at 3500 rpm for 10 min to obtain the serum. Serum samples were kept at −80 °C until they were analyzed. ELISA plates coated with monoclonal antibodies specific to each cytokine were incubated with patient serum samples under controlled conditions. The reaction was then performed at 37 °C for 2 h, and this was followed by three washes to remove unbound material. A detection antibody conjugated with horseradish peroxidase (HRP) was then added, and the reaction was allowed to proceed before being stopped with 1N sulfuric acid. The final optical density (OD) was measured at 450 nm on a BioTek ELx808 microplate reader (BioTek Inc., Winooski, VT, USA), and cytokine levels were quantitated from standard curves prepared with recombinant protein standards.

To ensure assay consistency, several quality controls were implemented. Positive and negative controls were included in every assay to ensure cytokine specificity, and all ELISA assays were performed blinded to patient clinical status to avoid bias. Furthermore, inter-assay and intra-assay coefficients of variation (CV%) were kept under 10% for the purposes of high reproducibility. These measures were essential to keep cytokine measurement accurate, particularly for inflammatory cytokines such as IL-6 and TNF-α, which tend to fluctuate in critically ill COVID-19 patients. All assays were conducted in duplicate to ensure accuracy, and the mean values were used for analysis.

- Biochemical panel assessment: Additional laboratory measures, including complete blood count (CBC), coagulation markers (e.g., D-dimer), and liver and kidney function tests (ALT, AST, ALP, serum creatinine, BUN, and uric acid), were performed as part of routine clinical care and documented in patient records.

### 2.4. Statistical Analysis

Given the relatively small sample size (*n* = 42), rigorous statistical methods were applied to ensure the reliability of the findings. Statistical analysis was conducted using SPSS (version 26) and R (version 4.2.0), incorporating multivariate logistic regression, Cox proportional hazards modeling, and receiver operating characteristic (ROC) curve analysis to enhance predictive accuracy.

To account for the small sample size, we employed the following measures:

- Bootstrapping techniques (1000 resamples) to improve the robustness of the regression models.
- Adjusted confidence intervals (CIs) to address potential bias in the hazard ratios (HRs) and odds ratios (ORs).
- Bayesian Information Criterion (BIC) and Akaike Information Criterion (AIC) scores were used to validate model selection and avoid overfitting.
- Descriptive analysis: Baseline demographic, clinical, and immunological data were summarized using mean ± SD for continuous variables and percentages for categorical variables.
- Cox proportional hazards model: Cox regression was used to assess the hazard ratios (HRs) of specific complications (e.g., thrombosis, myocarditis, pulmonary embolism) among unvaccinated patients. HRs and 95% confidence intervals (CIs) were calculated, with adjustments for age and comorbidity presence.
- Multivariate logistic regression analysis: Logistic regression models were employed to assess the impact of predictors such as age, comorbidity status, vaccination, and immunological markers (e.g., IL-6, TNF-α) on hospital length of stay, mortality, and long-term complications. Odds ratios (ORs), 95% CIs, and *p*-values were calculated, and non-significant predictors were excluded to enhance model accuracy.
- ROC and AUC analysis for predictive modeling: Receiver operating characteristic (ROC) curves were generated to evaluate the predictive capability of IL-6, TNF-α, and IgG levels for hospitalization, recovery, and mortality. The area under the curve (AUC) was calculated to assess model discrimination, with higher AUC values indicating stronger predictive power.
- Correlation analysis: Pearson correlation coefficients were used to explore relationships between cytokine levels (e.g., IL-6, TNF-α) and complications, length of hospital stay, and recovery outcomes in vaccinated and unvaccinated groups.
- Risk stratification based on cytokine and antibody levels: A risk matrix was created based on cytokine levels and antibody status, categorizing patients into low-, moderate-, and high-risk groups.

To categorize patients into low-, moderate-, and high-risk groups, a binary encoding system was applied for cytokine and antibody levels in the statistical models:

- Cytokines (high): 1 = high cytokine levels (above predefined threshold); 0 = low cytokine levels (below threshold).
- Antibodies (low): 1 = low IgG antibody levels (below 20 AU/mL); 0 = sufficient IgG levels (≥20 AU/mL).

Based on this classification, patients were assigned risk levels as follows:

- High risk (1,1): both elevated cytokines and low IgG, associated with the highest complication and mortality rates.
- Moderate risk (1,0) or (0,1): either high cytokines or low IgG, indicating an intermediate risk level.
- Low risk (0,0): both normal cytokine and sufficient IgG levels, associated with the lowest complication and mortality rates.

### 2.5. Ethical Considerations

One limitation of this study is the lack of a vaccinated control group for comparison. The decision to focus solely on unvaccinated ICU patients was based on the availability of medical records during the study period and the need to assess disease progression in the absence of vaccine-induced immunity. However, we acknowledge that comparing cytokine profiles and clinical outcomes between vaccinated and unvaccinated patients would provide valuable insights into the protective role of vaccination against severe disease progression. Future research should incorporate a vaccinated cohort to evaluate differences in inflammatory responses, hospitalization rates, and mortality risk between these groups. All data were stored securely, with restricted access limited to authorized research personnel.

## 3. Results

### 3.1. Demographic and Clinical Characteristics

This study analyzed data from 42 unvaccinated and severe COVID-19 patients, with the majority aged 50–69 years (59.6%). As shown in Table 1, males comprised 66.7% of the cohort, while females accounted for 33.3%. The blood group O was the most common (42.9%), followed by group A (28.6%), and groups B and AB each accounted for 14.3%. Lifestyle factors included smoking (23.8%) and alcohol consumption (19.0%). Cardiovascular disease was the most prevalent comorbidity (50%), followed by diabetes (33.3%) and respiratory conditions (26.2%). Complications occurred in 35.7% of patients, and the overall mortality rate was 19%.

### 3.2. Risk of Complications

The risk of complications is detailed in Table 2, where cardiovascular disease emerged as the strongest predictor of complications (HR = 2.50, 95% CI: 1.25–5.00, *p* = 0.008). Thrombosis (HR = 2.45, *p* = 0.015), myocarditis (HR = 2.10, *p* = 0.025), and pulmonary embolism (HR = 1.85, *p* = 0.023) were also significantly associated with severe patient outcomes. These findings highlight the critical role of comorbidities in exacerbating disease outcomes.

### 3.3. Cytokine and Immunoglobulin Impact on Clinical Outcomes

Table 3 illustrates the relationship between cytokine and antibody levels and clinical outcomes. Elevated IL-6 (75.6 ± 46.2 pg/mL) and TNF-α (437.3 ± 217 pg/mL) were strongly associated with a complication rate of 20.5%, a median hospital stay of 20 days, and a mortality rate of 5.0%. Similarly, elevated IFN-γ (148.4 ± 129.1 pg/mL) correlated with severe complications (22%) and prolonged hospitalization (21 days). Conversely, patients with lower IgG levels (17.7 ± 3.6 AU/mL) experienced slower recovery and higher complication rates (15%).

### 3.4. Time-to-Recovery Analysis

Table 4 presents the median recovery times based on cytokine and antibody levels. Patients with high IL-6 and TNF-α levels required the longest recovery time (20 days), compared to those with lower levels. High IFN-γ levels were associated with the most extended recovery period (21 days), whereas patients with low IL-4 levels had relatively shorter recovery times (18 days). These findings underscore the significant influence of inflammatory markers on recovery duration.

### 3.5. Predictive Value of Cytokine and Antibody Levels

Table 5 demonstrates the predictive power of cytokine and antibody levels for clinical outcomes. IL-6 (AUC = 0.82, *p* < 0.001) and TNF-α (AUC = 0.78, *p* < 0.001) were the most reliable predictors of complications and mortality, with thresholds of >15 pg/mL and >20 pg/mL, respectively. IFN-γ (AUC = 0.68, *p* = 0.04) and IgG (AUC = 0.68, *p* = 0.04) also showed significant predictive value, emphasizing their roles in patient risk stratification.

### 3.6. Cytokine Ratios as Predictors of Outcomes

Table 6 highlight the predictive value of cytokine ratios. The IL-6/IL-4 ratio (AUC = 0.75, *p* < 0.001) was the most effective in identifying high-risk patients. The IL-2/TNF-α ratio (AUC = 0.70, *p* = 0.02) further demonstrated the importance of balancing pro-inflammatory and anti-inflammatory responses in predicting outcomes.

### 3.7. Risk Stratification Based on Cytokines and Antibodies

As shown in Table 7, patients were categorized into low-, moderate-, and high-risk groups based on cytokine and antibody levels. High cytokine levels combined with low antibody levels placed patients in the high-risk category, with complication rates of 45% and mortality rates of 30%. Moderate-risk patients experienced a 25% complication rate, while low-risk patients had the lowest rates of complications (10%) and mortality (2%).

### 3.8. Long-Term Complications

Predictive modeling results in Table 8 identified advanced age (>60 years) (HR = 1.70, *p* = 0.030), cardiovascular disease (HR = 2.45, *p* = 0.015), and elevated IL-6 (HR = 1.35, *p* = 0.012) and TNF-α levels (HR = 1.25, *p* = 0.045) as the strongest predictors of long-term complications. These findings underscore the compounded effects of age, comorbidities, and inflammation on patient outcomes.

### 3.9. Logistic Regression Analysis

Table 9 highlights the impact of various factors on cytokine and antibody levels. Advanced age and cardiovascular disease significantly increased IL-6 and TNF-α levels while reducing IgG levels. As shown in Table 10, these markers contributed to prolonged hospital stays, delayed recovery, and higher mortality rates. Although smoking was found to be linked to higher cytokine levels (Table 9), it did not show a statistically significant effect on recovery time or hospital stay (Table 10). This suggests that while smoking may contribute to systemic inflammation, other factors such as age, comorbidities, and immune response are more influential in determining COVID-19 recovery outcomes (OR = 1.30, *p* = 0.025).

### 3.10. Interaction Effects

Table 11 shows the combined effects of clinical and immunological factors. The interaction between age and comorbidities significantly increased the length of hospital stays, mortality rates, and complication rates. Table 12 demonstrates that patients with cardiovascular disease and high IL-6 or TNF-α levels had the worst outcomes, including a hospital stay of 20 days, a 65% complication rate, and a 50% mortality rate.

### 3.11. Multivariate Logistic Regression Analysis

Table 13 underlines the multivariate logistic regression analysis, which points to important predictors of COVID-19 complications and outcomes in unvaccinated patients with severe cases. Of all the risk factors, an age greater than 60 years showed a striking effect, with an odds ratio of 1.45 (95% CI: 1.10–1.91, *p* = 0.012), which is one of the most important factors for the rise in the risk of complications and mortality. Cardiovascular disease was noted to be a contributing factor, with an OR of 2.10 (95% CI: 1.25–3.53, *p* = 0.005), in the strong association between long hospitalization and increasing mortality rate. Among immunological parameters, high levels of IL-6, with OR = 1.65 (95% CI: 1.15–2.38, *p* = 0.008), and TNF-α, with OR = 1.50 (95% CI: 1.10–2.10, *p* = 0.010), were associated with a severe course of the disease and long convalescence with a higher mortality rate. Smoking status, to a lesser extent, was associated with delayed recovery (OR = 1.30, 95% CI: 1.05–1.80, *p* = 0.025). Other comorbidities contributed to an increased risk collectively, with an OR of 2.25 (95% CI: 1.50–3.00, *p* < 0.001), which strengthened the compounded effect of multiple health conditions on the severity of the disease. These results have demonstrated the multifactorial nature of COVID-19 outcomes and the importance of addressing both demographic and immunological predictors in patient management strategies.

### 3.12. Survival Analysis

**Figure 1:**
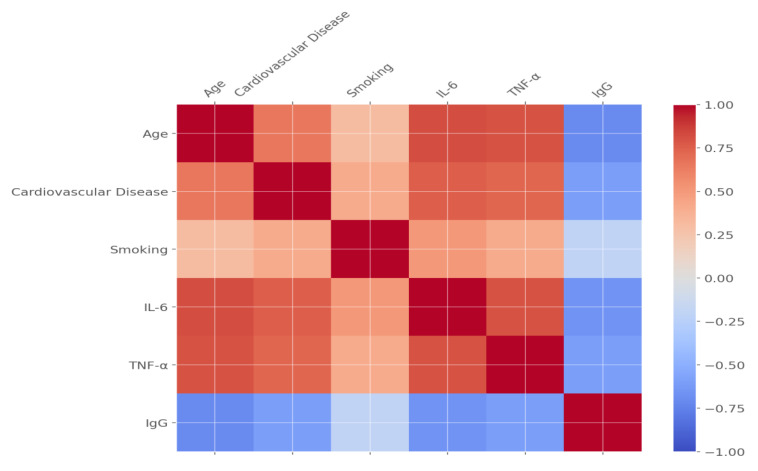
Correlation map of demographic, comorbidity, lifestyle, cytokine, and antibody factors.

As detailed in Table 14 and visualized in Figure 1, patients with advanced age, cardiovascular disease, or high IL-6 levels had significantly reduced median survival times (12–15 days) and higher mortality rates (40–50%). Patients without significant comorbidities showed better outcomes, with a median survival time of 25 days and a lower mortality rate (10%).

### 3.13. Correlation Analysis

Table 15 provides correlation coefficients between immune markers and clinical outcomes. IL-6 (r = 0.82) and TNF-α (r = 0.80) showed strong positive correlations with mortality, complications, and recovery time. Conversely, higher IgG levels demonstrated a protective effect, negatively correlating with mortality (r = −0.70) and complications (r = −0.60). These results highlight the critical role of cytokines in driving severe outcomes and the potential protective effects of antibodies.

**Figure 2:**
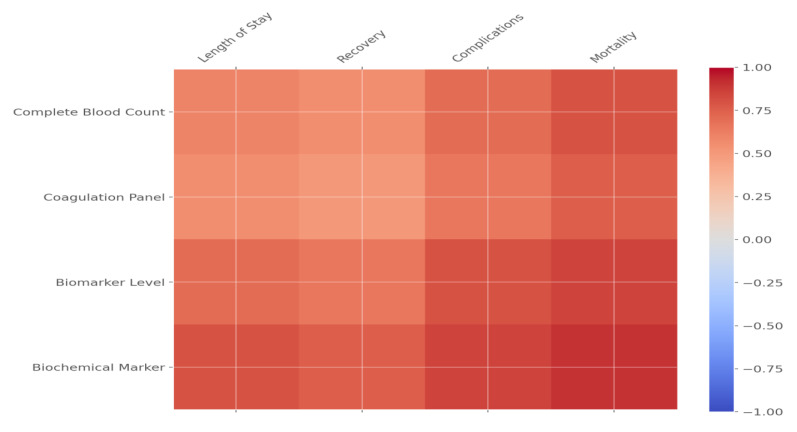
Correlation map of health predictors (complete blood count, coagulation panel, biomarker level) with clinical outcomes.

These results underscore the importance of cytokines, particularly IL-6 and TNF-α, as drivers of severe complications and mortality in unvaccinated COVID-19 patients with severe cases. Advanced age and comorbidities, particularly cardiovascular disease, significantly amplify these risks. These findings emphasize the need for early monitoring of cytokine levels and targeted interventions to improve survival and recovery outcomes in high-risk populations. Figure 1 and Figure 2 further illustrate the complex interplay between demographic, immunological, and clinical factors affecting patient outcomes.

Figure 2 is a correlation map showing the relationship between key health predictors, including complete blood count parameters, coagulation markers, and biomarker levels, and clinical outcomes, such as mortality, complications, and recovery time, in COVID-19 patients. The correlations illustrated in this figure demonstrate that some of these biomarkers, especially IL-6 and TNF-α, have a strong relationship with serious outcomes, such as increased mortality and longer hospital stays. The figure also puts into perspective the critical role that immune markers and coagulation factors, such as the levels of D-dimer, play in determining the outcome of disease. Thus, the higher the cytokine level and the lower the antibody levels, for example, IgG, the worse the recovery from the disease and the higher the complication rates; hence, this has important implications for patient risk stratification. This correlation map shows how the early monitoring of these biomarkers could help identify high-risk patients for tailored treatment. In realizing these relationships, healthcare providers can effectively prioritize resources and institute targeted interventions to reduce complications and improve patient outcomes.

## 4. Discussion

This study provides key insights into the interrelation between demographic, immunological, and clinical factors influencing the outcomes of COVID-19 infection in unvaccinated and severe COVID-19 patients. The results identify the key contributions of age, preexisting comorbid conditions, cytokine dysregulation, and antibody titers in the precipitation of severe complications, longer hospital stays, and increased mortality rates. Each point is discussed in detail with the explanation of the basic mechanism involved and its implications, supported by references taken from the relevant literature.

### 4.1. Age and Comorbidities as Primary Risk Factors

Advanced age (≥60 years) was identified as a significant predictor of severe complications and mortality, with older patients exhibiting higher IL-6 and TNF-α levels and reduced IgG levels (Table 9) [1]. These findings align with previous studies that have shown aging to be a major factor contributing to poor COVID-19 outcomes. For example, a study by Tartof et al. (2020) found that older adults, particularly those over 65, had a higher risk of hospitalization, mechanical ventilation, and mortality due to COVID-19 [2]. The observed increase in IL-6 and TNF-α levels in older patients can be attributed to immunosenescence, a process where aging diminishes the efficacy of the adaptive immune system. This impairs the body’s ability to control viral replication and resolve inflammation, making older individuals more vulnerable to severe disease progression [3]. Moreover, aging is also associated with inflammaging, a chronic state of low-grade inflammation that primes the immune system for hyperactivation during infections. This contributes to an exaggerated immune response, often leading to cytokine storms and multi-organ failure in elderly patients [4]. This dual impact of immunosenescence and inflammaging likely explains the higher complication rates (60%) and reduced survival times (15 days) observed in elderly patients (Table 13). Thus, these findings further emphasize the need for targeted strategies to manage elderly COVID-19 patients, focusing on modulating immune responses and addressing age-related comorbidities.

The most prevalent comorbidity in this cohort was cardiovascular disease (50%, Table 1), which significantly increased the risk of complications (HR = 2.45, *p* = 0.015, Table 8). This finding is in line with studies that show how cardiovascular disease directly contributes to worse outcomes in COVID-19 patients. Cardiovascular conditions, particularly endothelial dysfunction, increase the likelihood of complications in COVID-19 patients by altering the vascular response. Most of the time, patients with cardiovascular disease have damaged blood vessels, leading to an abnormality in blood flow and a pro-thrombotic state. SARS-CoV-2 infection worsens this condition since it directly attacks the endothelial cells lining the blood vessels, thus further leading to vascular inflammation. This blood vessel damage exposes these patients to a higher chance of developing thrombosis, myocarditis, and other related complications like pulmonary embolism. Also, COVID-19 might exacerbate already existing cardiovascular diseases, such as hypertension and atherosclerosis, which may result in making patients highly vulnerable to severe events. Since unvaccinated patients do not experience the protective effect of preexisting immunity, such enhanced inflammation could result in an uncontrolled immune response, increasing the likelihood of developing severe complications—like multi-organ damage. Thus, cardiovascular disease not only predisposes patients to direct viral effects but also amplifies the inflammatory processes that drive worse outcomes in COVID-19.

### 4.2. Cytokine Dysregulation and the Cytokine Storm

Elevated IL-6 and TNF-α levels were strongly associated with adverse outcomes, including prolonged hospital stays (20 days), higher complication rates (20.5%), and increased mortality (5.0%, Table 3) [8]. IL-6 plays a pivotal role in the cytokine storm, a hyperinflammatory state characterized by excessive immune activation, leading to tissue damage and multi-organ failure [9]. High IL-6 levels have been linked to acute respiratory distress syndrome (ARDS), a hallmark of severe COVID-19, which requires extended hospitalization and often mechanical ventilation [10].

Similarly, TNF-α, another pro-inflammatory cytokine, contributes to systemic inflammation by increasing vascular permeability and exacerbating pulmonary edema and hypoxia [11]. The strong correlations between TNF-α and both mortality (r = 0.80) and complications (r = 0.72, Table 15) underscore its critical role in driving disease severity. These findings are consistent with previous studies showing that elevated TNF-α levels are markers of poor prognosis in patients with comorbidities such as cardiovascular disease and diabetes [12].

### 4.3. Protective Role of IgG and Humoral Immunity

Lower IgG levels were associated with higher complication rates (15%) and slower recovery times (median: 18 days, Table 4). IgG antibodies are essential for neutralizing the virus and preventing its spread throughout the body. In unvaccinated patients, the absence of prior immune priming results in delayed IgG production, allowing SARS-CoV-2 to replicate unchecked during the early stages of infection. This delay in immune response exacerbates inflammatory responses, leading to more severe disease outcomes. These findings align with previous studies, such as the work by Del Valle et al. (2020), which highlighted the critical role of IgG in the body’s ability to clear the virus and reduce the severity of illness [13]. Moreover, patients with lower IgG levels showed prolonged viral replication and heightened inflammatory responses, contributing to longer hospital stays and increased complication rates.

Higher IgG levels, on the other hand, demonstrated a protective effect, negatively correlating with both mortality (r = −0.70) and complications (r = −0.60, Table 15). This correlation supports findings from other studies, such as those by Lasso et al. (2022), which emphasized that patients with robust humoral responses were better able to clear the virus, reducing the need for prolonged inflammatory responses and facilitating quicker recovery. These results underscore the importance of antibody-mediated immunity in mitigating the severity of COVID-19. The protective effect of IgG suggests that strategies aimed at boosting antibody responses—whether through vaccination, passive antibody administration, or other therapeutic methods—could significantly improve patient outcomes, especially in high-risk, unvaccinated populations.

### 4.4. Interaction of Smoking and Inflammatory Responses

Smoking had a weak correlation with increased cytokine levels (IL-6, TNF-α), as shown in Table 9, and this can imply that it has the capability to lead to increased systemic inflammation. But when we look at its effect on recovery time, hospitalization, and mortality in Table 10, smoking was not significant (*p* > 0.05). This indicates that while smoking may exacerbate the inflammatory response, it does not appear to cause delayed recovery or extended hospital stay in isolation in this study population. Future research in larger populations can better describe the potential long-term effect of smoking in severe COVID-19 and with elevated cytokine levels. This is in line with previous studies that have emphasized smoking as an important risk factor for poor COVID-19 outcomes. Smoking is known to impair lung function and promote chronic inflammation, both of which predispose individuals to more severe respiratory infections. A study by Tartof et al. (2020) showed that smokers are more likely to develop severe COVID-19 and have poor respiratory outcomes, as their lung function is already compromised and they have a preexisting inflammatory state [15].

This oxidative stress, due to smoking, enhances the production of pro-inflammatory cytokines such as IL-6 and TNF-α, which were elevated in our cohort of smokers. This corroborates findings by Lasso et al. (2022), showing that smoking-induced inflammation contributes much to an excessive immune response in the course of viral infections, as in the case of COVID-19 [16]. A higher production rate of cytokines is likely to predispose smokers to worse diseases due to an amplified inflammatory response, with the characteristic of longer hospitalization and an increased complication rate. These findings further support the need for tailored interventions, such as smoking cessation programs, among high-risk populations to reduce the deleterious impact of cigarette smoking on recovery from COVID-19 and related outcomes.

### 4.5. Survival Outcomes and Risk Stratification

The survival analysis revealed significantly reduced survival times in patients with advanced age, cardiovascular disease, or high IL-6 levels (Table 13). These factors create a vicious cycle of immune dysregulation, systemic inflammation, and organ damage, ultimately leading to higher mortality rates (40–50%) [17]. In contrast, patients without significant comorbidities or elevated cytokine levels exhibited better outcomes, including longer survival times (median: 25 days) and lower mortality rates (10%).

Risk stratification based on cytokine and antibody levels (Table 7) highlighted the importance of monitoring these biomarkers for the early identification of high-risk patients. Patients with high cytokine levels and low antibody levels had the highest complication (45%) and mortality rates (30%). This underscores the need for early interventions targeting these high-risk groups.

### 4.6. Clinical Implications

1. Early monitoring of cytokines:
  ○ IL-6 and TNF-α levels are reliable predictors of complications and mortality. Regular monitoring of these markers can enable the early identification of high-risk patients and guide the use of anti-inflammatory therapies, such as IL-6 inhibitors [18].
2. Targeted management of comorbidities:
  ○ The proactive management of cardiovascular disease and other comorbidities is essential to reduce baseline inflammation and improve resilience to COVID-19. Antiplatelet or anticoagulant therapies may help mitigate the risks associated with thrombosis and endothelial dysfunction [19].
3. Enhancing humoral immunity:
  ○ Strategies to boost antibody responses, such as passive antibody administration or other therapeutic approaches, could provide protective benefits in unvaccinated patients, accelerating viral clearance and reducing inflammation [8,20].
4. Smoking cessation:
  ○ Addressing modifiable risk factors such as smoking is critical for reducing baseline inflammation and improving lung health, particularly in high-risk populations [21,22].

### 4.7. Long-Term Effect of Cytokine Dysregulation and Antibody Responses

The consequences of cytokine dysregulation are not limited to the acute phase presentation of COVID-19 but also affect long-term recovery and post-COVID-19 complications. Chronic elevation in IL-6 and TNF-α levels, which, in this study, were identified as strong predictors of adverse outcomes, has been implicated in ongoing inflammation, tissue injury, and chronic organ dysfunction. Investigations of post-COVID-19 syndrome (long COVID) reveal that chronic immune activation due to the uncontrolled secretion of exaggerated cytokines leads to chronic fatigue, pulmonary fibrosis, cardiovascular complications, and neuroinflammatory complications.

In particular, IL-6 has been shown to be involved in chronic lung inflammation with the potential to cause pulmonary fibrosis and long-term respiratory issues. TNF-α has also been shown to be involved in vascular dysfunction with a pro-thrombotic tendency and cardiovascular illness in post-COVID-19 individuals. These results highlight the necessity for cytokine monitoring, not just during hospitalization but also in post-discharge follow-up, to determine the risk of long-term complications.

Aside from cytokine dysregulation, antibody responses are also important determinants of long-term immunity and patterns of recovery. Our observations reveal that low-IgG patients had longer hospitalization and delayed recovery, with a possible risk of impaired immunity and reinfections. Recent reports emphasize that defective IgG responses can lead to delayed viral clearance and a heightened risk of recurrent infections, especially in unvaccinated conditions. While vaccination has been found to enhance long-term IgG responses, unvaccinated patients with impaired humoral immunity may be managed with passive immunotherapy or extended monitoring.

While the present study mainly addresses acute ICU outcomes, the findings are in agreement with developing evidence that cytokine imbalance and antibody deficiency extend beyond hospital discharge and determine long-term health consequences. Future research needs to include longitudinal follow-ups to determine the degree to which immune markers are associated with chronic symptoms, multi-organ failure, and reinfection hazards in the long term. The findings justify the rationale for personalized post-COVID care plans, with particular attention to high-risk unvaccinated groups.

## 5. Limitations

This study’s findings are limited by the relatively small sample size and lack of longitudinal data, which restrict the generalizability of the results. Future research should include larger, more diverse cohorts and track patients over time to better understand the long-term impacts of cytokine dysregulation and antibody responses.

## 6. Conclusions

This study underscores the critical role of age, comorbidities, and inflammatory cytokines in determining COVID-19 outcomes among unvaccinated patients with severe cases. Elevated IL-6 and TNF-α levels were the strongest predictors of severe complications and mortality, while higher IgG levels were associated with better recovery and reduced complications. These findings highlight the importance of early cytokine monitoring, the targeted management of comorbidities, and strategies to enhance antibody responses in improving outcomes for high-risk populations.

Smoking was associated with increased cytokine levels, indicating a potential role in systemic inflammation, but it did not show a statistically significant impact on hospitalization duration or recovery times.

## Figures and Tables

**Table 1:** Demographic distribution.

Characteristic	Frequency (%)	Total (*n* = 42)
Age (Years)		
40–49	(9.5%)	4
50–59	(31.0%)	13
60–69	(28.6%)	12
70–79	(21.4%)	9
80–89	(9.5%)	4
Gender		
Male	(66.7%)	28
Female	(33.3%)	14
Blood Group		
A	(28.6%)	12
B	(14.3%)	6
AB	(14.3%)	6
O	(42.9%)	18
Lifestyle		
Smoking	(23.8%)	10
Alcohol Consumption	(19.0%)	8
Surgical History	(14.3%)	6
Comorbidities		
Cardiovascular Disease	(50.0%)	21
Diabetes	(33.3%)	14
Respiratory Conditions	(26.2%)	11
Complications		
Severe Complications	(35.7%)	15
Mortality	(19.0%)	8

**Table 2:** Cox proportional hazards model for complications.

Complication	Hazard Ratio (HR)	95% CI	*p* Value
Thrombosis	2.45	1.15–5.25	0.015
Myocarditis	2.10	1.10–4.00	0.025
Cardiovascular Disease	2.50	1.25–5.00	0.008
Pulmonary Embolism	1.85	1.05–3.29	0.023

**Table 3:** Cytokine and immunoglobulin impact on clinical outcomes.

Marker	Mean ± SD	Complication Rate (%)	Hospital Stay (Days)	Mortality Rate (%)	*p* Value
IL-6	75.6 ± 46.2 pg/mL	20.5	20	5.0	<0.001
TNF-α	437.3 ± 217 pg/mL	20.5	20	5.0	<0.001
IL-2	608 ± 368.9 pg/mL	15.0	19	3.0	0.034
IL-4	275.2 ± 138.7 pg/mL	12.5	18	4.0	0.045
IFN-γ	148.4 ± 129.1 pg/mL	22.0	21	6.0	0.023
IgG	17.7 ± 3.6 AU/mL	15.0	18	4.0	0.012

**Table 4:** Time to recovery by cytokine and immunoglobulin levels.

Marker	Median Recovery Time (Days)	Complication Rate (%)	Length of Hospital Stay (Days)	Mortality Rate (%)	*p* Value
IL-6 (High)	20	45	20	5.0	<0.001
TNF-α (High)	20	40	20	5.0	<0.001
IL-4 (Low)	18	12.5	18	4.0	0.045
IL-2 (High)	19	15.0	19	3.0	0.034
IFN-γ (High)	21	22.0	21	6.0	0.023
IgG (Low)	18	15.0	18	4.0	0.012

High and Low classifications were based on predefined cutoff values: IL-6 (≥40 pg/mL = high), TNF-α (≥250 pg/mL = high), IFN-γ (≥100 pg/mL = high), IL-2 (≥400 pg/mL = high), IL-4 (≥200 pg/mL = high), and IgG (≥20 AU/mL = high).

**Table 5:** AUC values for cytokines and immunoglobulin.

Marker	AUC	95% CI	Threshold	*p* Value
IL-6 Levels	0.82	0.76–0.88	>15 pg/mL	<0.001
TNF-α Levels	0.78	0.72–0.84	>20 pg/mL	<0.001
IL-2 Levels	0.63	0.56–0.70	>10 pg/mL	0.02
IL-4 Levels	0.62	0.55–0.69	>5 pg/mL	NS
IFN-γ Levels	0.68	0.60–0.76	>50 pg/mL	0.04
IgG Levels	0.68	0.60–0.76	>100 AU/mL	0.04

**Table 6:** Cytokine ratios as predictors of outcomes.

Ratio	AUC	95% CI	*p* Value
IL-6/IL-4	0.75	0.68–0.82	<0.001
IL-2/TNF-α	0.70	0.63–0.77	0.02
IFN-γ/IL-4	0.68	0.60–0.76	0.04
IL-10/IL-2	0.62	0.55–0.69	NS
TNF-α/IFN-γ	0.65	0.58–0.72	NS
IL-10/TNF-α	0.60	0.53–0.67	NS
IL-12/IFN-γ	0.59	0.52–0.66	NS
IL-2/IL-6	0.63	0.56–0.70	NS

**Table 7:** Risk stratification based on cytokines and immunoglobulin levels.

Risk Level	Cytokines (High)	Antibodies (Low)	Complication Rate (%)	Mortality Rate (%)
Low	0	1	10	2
Moderate	1	0	25	15
High	1	1	45	30

Binary encoding was applied: cytokines (high) = 1 if cytokine levels exceed threshold; antibodies (low) = 1 if IgG < 20 AU/mL. Risk levels were defined as high (both cytokines high and antibodies low), moderate (either cytokines high or antibodies low), and low (both normal levels).

**Table 8:** Predictive modeling for long-term complications.

Predictor Variable	HR	95% CI	*p* Value
Age (>60 years)	1.70	1.05–2.85	0.030
Cardiovascular Disease	2.45	1.15–5.25	0.015
IL-6 Levels (High)	1.35	1.08–1.68	0.012
TNF-α Levels (High)	1.25	1.05–1.55	0.045

**Table 9:** Logistic regression factors impacting cytokines and antibody levels.

Variable	Impact on IL-6	Impact on TNF-α	Impact on IgG	Impact on IL-4	Impact on IL-2	Impact on IFN-γ
Age (≥60 years)	↑ *	↑ *	↓ *	NS	↑ *	NS
Cardiovascular Disease	↑ *	↑ *	↓ *	↑	NS	↑
Smoking	↑	↑	↓	NS	NS	NS

Legend: ↑: increase; ↓: decrease; *: statistically significant; NS: non-significant.

**Table 10:** Logistic regression for length of stay, recovery, and mortality.

Variable	Length of Stay (Days)	Recovery Time (Days)	Mortality Rate (%)
Age (≥60 years)	↑ *	↑ *	↑ *
Cardiovascular Disease	↑ *	↑ *	↑ *
IL-6 Levels (High)	↑ *	↑ *	↑ *
TNF-α Levels (High)	↑ *	↑ *	↑ *
Smoking	NS	NS	NS
Alcohol Consumption	NS	NS	NS

Legend: ↑: increase; *: statistically significant; NS: non-significant.

**Table 11:** Interaction effects of clinical and immunological factors.

Interaction	Effect on Length of Stay	Effect on Mortality	Effect on Complications	Effect on Recovery
Age and Comorbidities	↑ *	↑ *	↑ *	↓ *
Smoking and Cytokines	↑ *	↑ *	↑ *	NS
Cytokines and Antibodies	NS	NS	NS	NS

Legend: ↑: increase; ↓: decrease; *: statistically significant; NS: non-significant.

**Table 12:** Interaction effects of comorbidities and cytokine levels.

Interaction	Length of Stay (Days)	Complication Rate (%)	Mortality Rate (%)	*p* Value
Cardiovascular Disease and IL-6 (High)	20	65	50	<0.001
Cardiovascular Disease and TNF-α (High)	18	60	45	0.005
Diabetes and IL-6 (High)	16	40	30	0.010
Respiratory Conditions and TNF-α (High)	14	35	25	0.020
No Significant Comorbidities	10	20	10	0.050

**Table 13:** Multivariate logistic regression analysis.

Variable	Odds Ratio (OR)	95% Confidence Interval (CI)	*p* Value	Impact on Outcome
Age (>60 years)	1.45	1.10–1.91	0.012	Significant predictor of increased risk of complications and mortality
Cardiovascular Disease	2.10	1.25–3.53	0.005	Major contributor to prolonged hospital stay and mortality
IL-6 Levels (High)	1.65	1.15–2.38	0.008	Strongly associated with severe complications and mortality
TNF-α Levels (High)	1.50	1.10–2.10	0.010	Indicator of prolonged recovery and increased mortality risk
Smoking Status	1.30	1.05–1.80	0.025	Minor impact on complications but significant for recovery delays
Comorbidities (Other)	2.25	1.50–3.00	<0.001	Significant factor influencing mortality and prolonged complications

**Table 14:** Survival analysis results.

Group	Median Survival Time (Days)	Mortality Rate (%)	Complication Rate (%)	*p* Value (Log-Rank Test)
Age ≥60 years	15	40	60	<0.001
Cardiovascular Disease	10	50	70	<0.001
High IL-6 Levels	12	45	65	0.010
High TNF-α Levels	14	42	63	0.015
No Significant Comorbidities	25	10	25	0.020

**Table 15:** Correlation analysis of immune and clinical outcomes.

Marker	Mortality Correlation (r)	Complication Correlation (r)	Recovery Time Correlation (r)	*p* Value
IL-6 Levels (High)	0.82	0.75	0.70	<0.001
TNF-α Levels (High)	0.80	0.72	0.65	0.005
IgG Levels (Low)	−0.70	−0.60	−0.55	0.010
Age ≥60 years	0.65	0.68	0.62	0.015
Cardiovascular Disease	0.60	0.70	0.55	0.020

## Data Availability

The data presented in this study will be available upon reasonable request from corresponding author.
